# Supplementary material for: Cannabidiol sensitizes TRPV2 channels to activation by 2-APB
Source: eLife. 2023 May 18;12:e86166. doi: 10.7554/eLife.86166 (PMC10195083; doi:10.7554/eLife.86166)
Supplement: Figure 3—figure supplement 1—source data 1. [file elife-86166-fig3-figsupp1-data1.pdf]

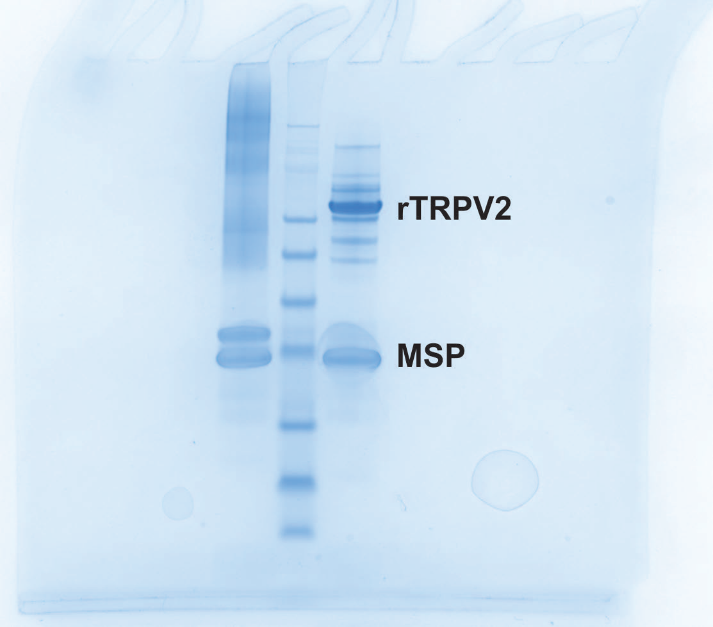
rTRPV2
MSP
